# Immune Checkpoint Inhibitor in First-Line Treatment of Metastatic Renal Cell Carcinoma: A Review of Current Evidence and Future Directions

**DOI:** 10.3389/fonc.2021.707214

**Published:** 2021-08-30

**Authors:** Iris Tung, Arvind Sahu

**Affiliations:** Department of Oncology, Goulburn Valley Health, Shepparton, VIC, Australia

**Keywords:** metastatic renal carcinoma, immune checkpoint inhibition (ICI), anti-VEGF (vascular endothelial growth factor) agents, tyrosine kinase inhibitions (TKIs) therapy, biomarker

## Abstract

The incidence of renal cell carcinoma (RCC) is rising and metastatic RCC carries a very poor prognosis. The treatment paradigm for metastatic RCC has shifted dramatically in the last decade with multi-targeted tyrosine kinase inhibitors (TKI) previously used as first-line treatment but its utility is limited by short-lived efficacy and rapid disease progression. The dysregulation of immune cells in the tumour microenvironment contributes to unregulated growth of RCC. Thus, the use of immune checkpoint inhibitors has become first-line treatment for metastatic RCC and has offered dramatic improvement in clinical benefit and survival. Treatment with immune checkpoint inhibitor in combination with TKI appears to be promising in offering even greater response rates. The treatment for metastatic RCC continues to evolve and ongoing advances with new targeted agents and biomarkers are needed to continue to improve prognosis in the future.

## Introduction

Renal cell carcinoma (RCC) has an incidence of approximately 400,000 cases per year globally, which is highest in North America, Europe and Australia (1). The incidence of RCC is rising over the last 50 years, which is attributable to increasing detection on imaging and increasing exposure to risk factors including obesity and alcohol consumption, particularly in developed countries (2).

The prognosis of RCC is poor as 30% of patients have metastatic disease at diagnosis with a 5-year survival rate of only 12% (3). Clear cell RCC (ccRCC) is the most common histological subtype and accounts for over 75% of RCCs, in comparison to non-clear cell RCC (nccRCC), which consists of 15 histological subtypes, including papillary and chromophobe histology (4). Prognosis can be conferred using the International Metastatic Renal Cell Carcinoma Database (IMDC), which may be used to assess risk in individual patients and can guide treatment decisions (5). Factors included in the IMDC are anaemia, neutrophilia, thrombocytosis, hypercalcaemia, Karnofsky performance status of less than 80 and less than 1 year from diagnosis to first-line systemic therapy (5). The presence of brain, bone or liver metastasis as the first site of metastatic disease prior to treatment was identified as a newly validated prognostic factor, which was associated with worse overall survival in the groups with favourable and intermediate IMDC risk (6).

**Figure 1:**
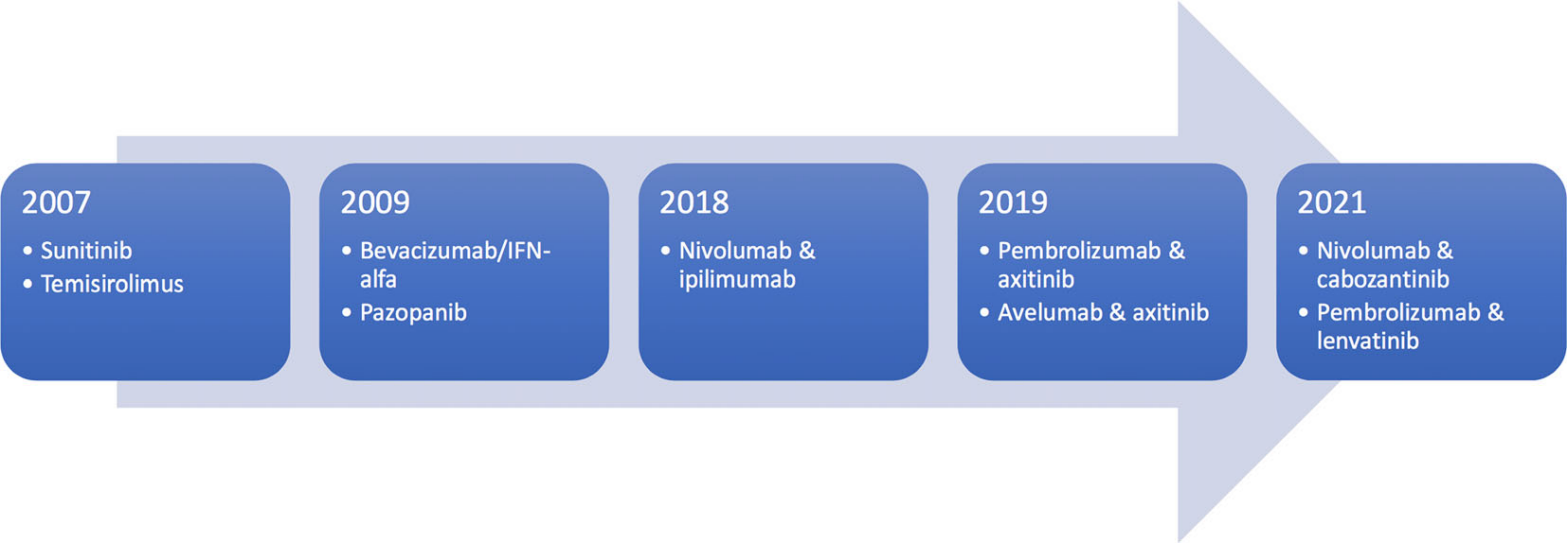
Timeline of FDA-approved treatment for metastatic RCC in first-line setting.

The advent of targeted treatment such as tyrosine kinase inhibitors improved survival outcomes for patients with metastatic RCC in the last decade (7). However, more recently, the use of immune checkpoint inhibitors has offered further improvement in outcomes for patients. This has dramatically altered the treatment paradigm for metastatic RCC and immune checkpoint inhibitors are increasingly used as the first-line treatment for metastatic ccRCC (**Figure 1**). This review will discuss the mechanism of immune checkpoint inhibitor in treatment of metastatic RCC, the key evidence supporting its use as first-line treatment and future research directions.

## Molecular Pathogenesis of RCC

The development of RCC is underpinned by abnormal angiogenesis. The von Hippel-Lindau (VHL) gene is a tumour suppressor gene that regulates activity of hypoxia-induced factor (HIF) and expression of vascular endothelial growth factor (VEGF) and platelet-derived growth factor (PDGF) (8). VHL is dysfunctional or inactivated in over 80% of ccRCC, resulting in increased HIF activity and overexpression of VEGF and PDGF which contributes to uncontrolled angiogenesis and tumour growth (8). Tyrosine kinase inhibitors (TKI) that inhibit VEGF pathway are anti-angiogenic and suppress tumour growth, with demonstrated efficacy in treatment of RCC (9). Anti-VEGF TKIs including sunitinib and pazopanib were previously used as first-line treatment of metastatic RCC. However, despite its initial efficacy, anti-tumour response is short-lived and tumour resistance inevitably develops during TKI treatment (10).

RCC is highly immunogenic and contributes to mobilisation of immune cells such as Tumour Infiltrating Lymphocytes (TILs) and natural killer cells into the tumour microenvironment, which promotes tumour growth (11, 12). Further, Programmed Death Ligand 1 (PD-L1) is widely expressed in RCC, which illustrates the importance of the PDL-1/PDL1 checkpoint in regulating tumour growth in RCC (12). Overexpression of PDL1 and its interaction with inhibitory PD-1 receptors results in downregulation and anergy of T cells, therefore downregulating host immune response against RCC (12–14). Immune checkpoint inhibitors including PD-1 inhibitors, pembrolizumab and nivolumab promote a long-lasting host immune response against tumour growth by inhibiting tumour-induced downregulation of host T cells (14).

## Immune Checkpoint Inhibitor Treatment in Metastatic ccRCC

The use of immune checkpoint inhibitors nivolumab and ipilimumab is now approved for first-line treatment of intermediate and poor-risk metastatic RCC and has demonstrated improved overall survival across multiple clinical trials (**Table 1**). Nivolumab is a PD-1 inhibitor which blocks the interaction of PD-1 on T cells with PD-L1, thereby preventing T cell inactivation (14). Nivolumab demonstrated anti-tumour activity and efficacy in a phase II trial in second-treatment of metastatic ccRCC, that had been previously treated with an anti-angiogenic agent (24). The objective response rate of nivolumab was approximately 20% and pleasingly 40% of responders had durable responses at 24 months (24). In the phase III trial, CheckMate-025, nivolumab used in a second-line treatment setting, demonstrated a higher objective response rate of 25% compared to 5% in everolimus and a significant increase in overall survival of 25 months compared to 19.6 months in the everolimus group (25).

**Table 1:** Summary of key phase III trials in the use of immune checkpoint inhibitors in first-line treatment of metastatic RCC.

Phase III Trial	Intervention	Control	Histology	Objective response rate	Progression Free Survival	Overall Survival
CheckMate 214, 2018 (15, 16)	Nivolumab (3mg/kg) & ipilimumab 1mg/kg) followed by nivolumab 3mg/kg every 2 weeks	Sunitinib 50mg daily for 4 weeks on, 2 weeks off	Clear cell	*Fav IMDC risk:* 29.6 *vs*. 51.6% p=0.0005 *Intermediate & poor IMDC risk:* 41.9 *vs*. 26.8% p<0.0001 *ITT:* 39.1 *vs*. 32.4 p=0.0134	*Fav IMDC risk:* 12.4 *vs*. 28.9 months HR 1.84 95% CI (1.29-2.62) *Intermediate & poor IMDC risk:* 11.2 *vs*. 8.3 months HR 0.74 95% CI (0.62-0.88) *ITT:* 12.2 *vs* 12.3 months HR 0.89 95% CI (0.76-1.05)	*Fav IMDC risk:* HR 0.93 95% CI 0.62-1.4 OS not reached *Intermediate & poor IMDC risk:* 48.1 *vs*. 26.6 months 50% *vs*. 35.8% HR 0.65 95% CI (0.54-0.78) *ITT:* 46.7 *vs*. 38.4 months 53.4% *vs*. 43.3% HR 0.69 95% CI (0.59-0.81)
JAVELIN Renal 101, March 2019 (17, 18)	Avelumab (10mg/kg) & axitinib 5mg twice daily	Sunitinib 50mg daily for 4 weeks on, 2 weeks off	Clear cell	51.4% *vs*. 25.7% p value not available	13.3 *vs*. 8.0 months p<0.0001	HR 0.796 95% CI 0.616-1.027 p=0.0392 (did not reach pre-specified significance level)
KEYNOTE-426, March 2019 (19, 20)	Pembrolizumab 200mg & axitinib 5mg twice daily	Sunitinib 50mg daily for 4 weeks on, 2 weeks off	Clear cell	59.3% *vs*. 35.7% p< 0.001	15.4 *vs*. 11.1 months p<0.0001	HR 0.68 95% CI 0.55-0.85 p=0.0003 Median OS not reached
IMmotion151, May 2019 (21)	Atezolizumab 1200mg & bevacizumab 15mg/kg	Sunitinib 50mg daily for 4 weeks on, 2 weeks off	Clear cell Sarcomatoid allowed	43% *vs*. 25% p value not available	11.2 *vs*. 8.4 months p=0.0219	63% *vs*. 60% at 24 months p=0.4751
CheckMate-9ER 2020 (22)	Nivolumab 240mg & cabozantinib 40mg daily	Sunitinib 50mg daily for 4 weeks on, 2 weeks off	Clear cell Sarcomatoid allowed	55.7% *vs*. 27.1% p<0.0001	16.6 *vs*. 8.3 months HR 0.51 95% CI 0.41-0.64 p<0.0001	85.7% *vs*. 75.6% at 12 months HR 0.6, 98% CI 0.4-0.89 P=0.001
CLEAR, 2021 (23)	*Arm A:* lenvatinib & pembrolizumab *Arm B:* lenvatinib & everolimus	Sunitinib 50mg daily for 4 weeks on, 2 weeks off	Clear cell Sarcomatoid allowed	71% *vs*. 53.5% *vs*. 36.1% p value not available	*Arm A *vs*. control:* 23.9 *vs*. 9.2 months p<0.001 *Arm B *vs*. control:* 14.7 *vs*. 9.2 months p <0.01	*Arm A *vs*. control:* HR 0.66 95% CI 0.49-0.88 p=0.005 OS not reached *Arm B *vs*. control:* HR 1.15 95% CI 0.88-1.5 p=0.3 OS not reached

In the pivotal phase III trial CheckMate-214, treatment with nivolumab and ipilimumab in the first-line setting for metastatic ccRCC resulted in a higher response rate (42% *vs*. 27%, p<0.001), progression-free survival and a significant increase in 12-month overall survival rate (80% *vs*. 72%, p<0.001), when compared to the control arm of sunitinib in those with intermediate or poor IMDC risk (15). The higher response rate and overall survival benefit offered by nivolumab and ipilimumab in the groups with intermediate and poor IMDC risk was ongoing after 4 years of follow up, demonstrating a durable response, with a 4-year overall survival rate of 50% compared to 35.8% in the control arm (16). Moreover, 10% of patients achieved complete response in the intervention arm across all IMDC risk groups, whereas treatment with sunitinib only offered a complete response rate of 1.4% and 6.5% in the intermediate to poor risk and favourable risk groups respectively (16). However, PD-L1 expression did not predict treatment response and survival benefit was observed independent of PD-L1 expression. Despite the use of two immune checkpoint inhibitors, there was a lower incidence of grade 3 and 4 treatment-related toxicities observed in the intervention arm in comparison to the use of sunitinib. Toxicities from nivolumab and ipilimumab were similar to that observed in immune checkpoint inhibitor studies in other solid organ malignancies, the most common of which included fatigue, pruritus, diarrhoea, rash and nausea. However, the incidence of grade 3 or above toxicities was still high at 46%. High dose corticosteroid treatment was required in 36% of patients experiencing toxicities, higher than when compared to treatment with immune checkpoint inhibitor combined with an anti-VEGF agent. Nonetheless, patient-reported quality of life was higher in the those who received immune checkpoint inhibitor treatment compared to sunitinib. This trial was practice-changing as immune checkpoint inhibitor with nivolumab and ipilimumab became the new standard-of-care first-line treatment for intermediate or poor risk metastatic RCC and was approved by the FDA in April 2018 for this indication.

More recently, Keynote-427, a phase II study investigated the efficacy of single-agent pembrolizumab in treatment-naïve metastatic RCC. Cohort A of this study recruited patients with ccRCC and results demonstrated efficacy of pembrolizumab with an objective response rate of 36.4%, progression free and overall survival rates of 22.3% and 70.8% respectively at 24 months of follow up. This benefit was observed regardless of PD-L1 expression and IMDC risk. The incidence of grade 3 or above toxicity was 30%, the most common of which was colitis. High dose corticosteroid treatment was required in 44% of cases of immune-related toxicities. Pembrolizumab may be a possible treatment option to TKI in those with favourable-risk disease with manageable toxicities. However, this study is limited by its single-arm design, therefore a phase III trial would be required to compare its efficacy and safety with sunitinib or ipilimumab with nivolumab (26).

## Immune Checkpoint Inhibitor & Anti-VEGF Treatment in Metastatic ccRCC

More recently, there is emerging evidence to support the use of immune checkpoint inhibitor in combination with anti-VEGF targeted agents for treatment of metastatic RCC in the first-line setting (**Table 1**). Anti-VEGF agents are important in their role in anti-angiogenesis, it is hypothesised that these agents are also important in moderating the immune system by promoting trafficking of immune cells to tumour microenvironment (27). Therefore, it is proposed that the combination of immune checkpoint inhibitor with anti-VEGF agents would act synergistically in reducing tumour burden.

The phase III trial JAVELIN Renal 101 demonstrated efficacy of PD-L1 inhibitor avelumab in combination with anti-VEGF agent axitinib in treatment-naive metastatic ccRCC (17). Treatment with avelumab and axitinib was associated with a higher response rate (51.4% *vs*. 25.7%) and a significantly higher progression-free survival (13.3 *vs*. 8 months, p< 0.0001) in comparison to the control arm of sunitinib. This benefit was observed regardless of PD-L1 level and IMDC risk. However, avelumab and axitinib did not offer a significant overall survival benefit compared to the control arm in an updated analysis in 2020 (18). Common toxicities associated with avelumab and axitinib include hypertension and skin toxicity but hepatotoxicity was more prevalent in the sunitinib group. Nonetheless, the FDA approved the use of avelumab and axitinib as first-line treatment for metastatic RCC in 2019.

The phase III trial, Keynote-426 delivered promising results for the use of PD-1 inhibitor pembrolizumab and anti-VEGF agent axitinib in first-line treatment for metastatic ccRCC (19, 20, 28). Treatment with pembrolizumab and axitinib demonstrated a significantly higher objective response rate (59.3% *vs*. 35.7%, p< 0.001), progression-free survival (15.4 *vs*. 11.1 months, p<0.0001) and overall survival (HR 0.68, 95% CI [0.55-0.85] p=0.0003) in comparison to sunitinib. This benefit was observed regardless of PD-L1 expression or IMDC risk. There were no unexpected treatment toxicities but there was a higher incidence of hepatotoxicity and rates of treatment discontinuation in the intervention arm. Hypertension and diarrhoea were common toxicities in both groups. Treatment with pembrolizumab and axitinib appears to offer durable anti-tumour response at long-term follow up with an objective response rate of 85%, progression-free survival and overall survival rates of 94.7% and 74.8% at 36 months respectively (28). Results from Keynote-426 are practice-changing as the combination of pembrolizumab and axitinib was approved by the FDA in April 2019 for first-line treatment of metastatic RCC.

The phase III trial, IMmotion151 included patients with ccRCC with sarcomatoid differentiation, which accounted for 16% of the study population. In this trial, first-line treatment with PD-L1 inhibitor atezolizumab and anti-VEGF monoclonal antibody bevacizumab was associated with a higher response rate (43% *vs*. 25%) and significant improvement in progression-free survival (11.2 *vs*. 8.4 months, p=0.02) compared to sunitinib but this did not translate into an overall survival benefit (21). Treatment with atezolizumab and bevacizumab was well tolerated with a lower incidence of grade 3 or more toxicities and rates of treatment discontinuation compared to sunitinib. Immune-related toxicities from immune checkpoint inhibitor were as expected, however with the addition of bevacizumab-related toxicities including hypertension and proteinuria. Despite a benefit in overall survival was not observed in this trial, this treatment appears to have activity in the group of ccRCC with sarcomatoid differentiation on subgroup analyses. This treatment regimen has not been granted FDA approval.

The phase III trial, Checkmate-9ER included patients with ccRCC with sarcomatoid differentiation, which constituted 11.5% of the study population and investigated the role of nivolumab and cabozantinib, a second-generation anti-VEGF agent, in treatment-naïve advanced ccRCC (22). Results from this trial are encouraging, treatment with nivolumab and cabozantinib was associated with significantly higher response rate (55.7% *vs*. 27.1%, p<0.0001), longer progression-free survival (16.6 *vs*. 8.3 months, p<0.0001) and 12-month overall survival (85.7% *vs*. 75.6%, p=0.001) compared to sunitinib. This benefit was observed across all subgroups including the group with ccRCC with sarcomatoid differentiation. Survival benefit was observed independent of PD-L1 expression and IMDC risk. No unexpected treatment-related adverse events were identified although rates of hepatotoxicity were higher in the intervention group. 19% of patients in the intervention arm required high dose corticosteroid treatment due to immune-related toxicities. However, patient-reported quality of life was greater in the intervention arm compared to sunitinib. First-line treatment with nivolumab and cabozantinib for metastatic RCC was most recently FDA-approved in January 2021 based on results from this trial. However, follow-up duration in this trial is reasonably short at 18 months and therefore durability of treatment response will need to be assessed at long-term follow up.

The phase III trial, CLEAR has recently been completed and investigated the efficacy of anti-VEGFR TKI lenvatinib either in combination with everolimus alone or combined with both everolimus and pembrolizumab in treatment-naïve ccRCC (23). This trial also included patients with ccRCC with sarcomatoid differentiation, which constituted approximately 20% of the study population. Treatment with lenvatinib and pembrolizumab was associated with a higher objective response rate (71% *vs*. 53.5% *vs*. 36.1%) compared to lenvatinib with everolimus and sunitinib respectively. Lenvatinib and pembrolizumab offered significantly higher progression-free survival (23.9 *vs*. 9.2 months, p<0.001) and higher overall survival (HR 0.66, 95% CI 0.49-0.88, p=0.005, OS NR) when compared to sunitinib. This benefit was observed regardless of PD-L1 level or IMDC risk. Lenvatinib and everolimus also offered longer progression-free survival (14.7 *vs*. 0.2 months, p<0.001) compared to sunitinib, but this did not translate into an overall survival benefit. However, toxicity appears to be an issue in this treatment and commonly included hypertension, diarrhoea, elevated lipase and hypertriglyceridaemia. 68.8% of patients in the lenvatinib and pembrolizumab group required dose reduction of lenvatinib and 37.2% of patients discontinued treatment as a result of toxicities. The FDA approved the use of lenvatinib and pembrolizumab in 2021 for treatment of metastatic RCC in 2021. Nonetheless, longer follow up data is required to continue to assess the efficacy and durability of response in this treatment.

## Discussion & Future Directions

### The Selection of First-Line Treatment Regimen in Metastatic ccRCC

The advent of immune checkpoint inhibitors has led to a plethora of new treatment options for metastatic RCC. The approach of combining immune checkpoint inhibitor with a TKI as first-line treatment of metastatic RCC appears promising, yielding higher response rates and improved survival outcomes, demonstrated across multiple phase III trials (**Table 1**). This is supported by the most recent National Comprehensive Cancer Network (NCCN) and European Association of Urology (EAU) guidelines in 2021, which both recommend first-line treatment with immune checkpoint inhibitor in combination with TKI regardless of IMDC risk or alternatively nivolumab and ipilimumab in intermediate and poor IMDC risk (17–23, 27–30). However, most clinical trials compared the efficacy of immune checkpoint inhibitors with sunitinib as the control, which is no longer considered the standard-of-care treatment.

There is no head-to-head trial evidence to compare the efficacy of the various treatment options available including immune checkpoint inhibitors, anti-VEGF therapy or a combination of both. There are multiple factors to consider when selecting first-line treatment for metastatic RCC. The IMDC prognostic risk model remains important in guiding selection of treatment. In favourable-risk disease, first-line treatment options include an anti-angiogenic agent alone or in combination with an immune checkpoint inhibitor, the latter option is favoured as illustrated in both NCCN and EAU guidelines in 2021. In favourable-risk disease, treatment with immune checkpoint inhibitor with TKI offers higher response rate and improved survival outcomes, when compared to treatment with sunitinib alone. In intermediate or poor risk disease, treatment options include ipilimumab and nivolumab or combining an immune checkpoint inhibitor with a TKI. Treatment with immune checkpoint inhibitor and TKI may be favoured in patients who are highly symptomatic with high disease burden and a rapid treatment response is desired, which may be offered by the TKI component of this treatment. Durability of treatment response should also be considered as there is now long-term follow up data to demonstrate the durable response and survival benefits offered by treatment with ipilimumab and nivolumab. In contrast, most clinical trials investigating various treatment regimens with immune checkpoint inhibitor and TKI have shorter follow up and immature long-term data, therefore it is unclear whether this treatment also offers similar durable responses when compared to ipilimumab and nivolumab. Toxicity is also an important consideration given higher rates of immune-related toxicities and requirement for high dose corticosteroid treatment associated with ipilimumab and nivolumab treatment compared to treatment with immune checkpoint inhibitor and TKI.

There are now multiple FDA-approved immune checkpoint inhibitor and anti-VEGF treatment regimens available, which further complicates the decision-making process in selecting treatment for patients with treatment-naïve RCC (**Figure 1**). The regimens used in JAVELIN Renal 101 and IMmotion 151 are unlikely to be preferred options given the lack of overall survival benefit and the latter is not FDA-approved. The treatment regimens used in Keynote-426, Checkmate-9ER and CLEAR all demonstrated impressive response rates but all had various issues with toxicity, and selection should be based on patient characteristics and their other co-morbidities. Lenvatinib and pembrolizumab treatment was associated with higher rates of hypertension and hyperlipidaemia, which may be an issue in patients with cardiovascular co-morbidities. Rates of hepatotoxicity were high in treatment with pembrolizumab with axitinib and nivolumab with cabozantinib, which may be challenging to manage in patients with underlying hepatic impairment. Secondly, histopathological features may guide decision-making as patients with ccRCC with sarcomatoid differentiation were only included in Checkmate-9ER and CLEAR and appear to derive benefit from treatment. Lastly, cost and access to treatment must be considered, which varies internationally. In Australia, only ipilimumab and nivolumab treatment is funded under the Pharmaceutical Benefit Scheme (PBS), none of the treatment regimens with immune checkpoint inhibitor and TKIs are available under PBS access at present.

### Current Clinical Trials Investigating Treatment Options in Metastatic ccRCC

The treatment landscape in metastatic RCC continues to evolve with multiple clinical trials investigating the role of combining immune checkpoint inhibitor with targeted agents in both treatment-naïve and treatment-refractory ccRCC (31), summarised in **Table 2**.The current active phase III clinical trials COSMIC-313 and PDIGREE use ipilimumab and nivolumab as the control arm unlike many previous trials which have historically used sunitinib as the control arm. The role of novel targeted agents is investigated in various phase I trials in heavily pre-treated RCC, including ciforadenant, an inhibitor of adenosine A2A receptor, which is expressed on T lymphocytes [NCT02655822].

**Table 2:** Current clinical trials investigating the use of immune checkpoint inhibitor with targeted agents in metastatic RCC.

NCT number	Phase	Histology	Intervention	Control	Primary Endpoint	Treatment Setting	Status
NCT03937219 (COSMIC 313)	III	Clear cell	Nivolumab & ipilimumab + cabozantinib	Nivolumab & ipilimumab only	PFS	First line	Recruiting
NCT03729245	III	Clear cell	Bempegaldesleukin & nivolumab	Sunitinib or cabozantinib	ORR, OS	First line	Recruiting
NCT03873402	III	Clear cell	Nivolumab & ipilimumab	Nivolumab alone	ORR, PFS	First line	Active, not recruiting
NCT04394975	III	Clear cell	Toripalimab & axitinib	Sunitinib	PFS	First line	Recruiting
NCT03260894	III	Clear cell	Pembrolizumab & epacadostat	Sunitinib or pazopanib	ORR	First line	Active, not recruiting
NCT03793166 (PDIGREE)	III	Clear cell	Ipilimumab & nivolumab followed by maintenance nivolumab & cabozantinib	Ipilimumab & nivolumab followed by maintenance nivolumab only	OS	First line	Recruiting
NCT03289962	I	Multiple cancers including ccRCC	Autogene cevumeran & atezolizumab	NA	DLT RP2D Adverse events	Subsequent line	Recruiting
NCT02964013	I	Multiple cancers including ccRCC	Vibostolimab (Anti-TIGIT antibody) & pembrolizumab	NA	DLT Adverse events	Subsequent line	Recruiting
NCT02655822	I	ccRCC	Ciforadenant (A2AR inhibitor) & atezolizumab	NA	DLT ORR Adverse events	Subsequent line	Recruiting
NCT02754141	I	Multiple cancers including ccRCC	BMS-986179 (CD73 inhibitor) & nivolumab	NA	Adverse events	Subsequent line	Recruiting

OS, overall survival; PFS, Progression-free survival; ORR, Objective response rate; DLT, Dose-Limiting Toxicites; RP2D, Recommended Phase 2 Dose.

Tivozanib is a selective and potent TKI that targets the VEGF receptor and demonstrated efficacy in treatment-naive RCC but did not show an overall survival benefit when compared to sorafenib in the phase III trial, TIVO-I (32). Similarly, the phase III trial, TIVO-III demonstrated an improved progression-free survival when tivozanib is used in heavily pre-treated patients with progressive RCC, but this did not translate into an overall survival benefit (33). More recently, the phase I/II trial TiNivo showed that treatment with tivozanib and nivolumab had a higher response rate of 56%, when compared to tivozanib alone (34). The phase III trial, TiNivo-2 is recruiting at present and aims to explore the progression-free survival and overall survival of treatment with tivozanib and nivolumab compared to tivozanib alone in previously treated patients with progressive RCC (35).

Hypoxia-inducible factor-2α (HIF-2α) accumulates abnormally in VHL inactivation, which results in tumour growth and progressive clear-cell RCC (8). Belzutifan is an HIF-2α inhibitor, which demonstrated activity in heavily pre-treated clear-cell RCC in a phase I trial, with an objective response rate of 25% (36). Toxicities included anaemia and hypoxia. The efficacy of belzutifan with cabozantinib is currently investigated in a phase II trial, which is recruiting both treatment-naïve patients and those who progressed with prior immune checkpoint inhibitor treatment [NCT03634540].

Indoleamine 2,3-dioxygenase 1 (IDO1) mediates anergy of effector T cells and contributes to the immunosuppressive tumour microenvironment (37). Therefore, the inhibition of IDO1 is hypothesised to prevent tumour-induced inhibition of T cell activation (37). Epacadostat is an IDO1 inihibitor which demonstrated anti-tumour activity when used with pembrolizumab in a phase I/II trial (38). Unfortunately, this treatment did not demonstrate progression-free or overall survival benefit when used to treat advanced melanoma in a phase III trial (39).

### The Search for New Biomarkers in Metastatic RCC

The identification of new predictive biomarkers and treatment targets is important to continue to improve the treatment of metastatic RCC. It has been demonstrated in many pivotal phase III clinical trials that PD-L1 expression is not a predictive biomarker as patients with negative PD-L1 expression also benefit from immune checkpoint inhibitor treatment. This is likely due to variable PD-L1 expression across different metastatic sites (4). However, PD-L1 expression may be a negative prognostic factor and was found to be associated with higher risk of death (40). PD-L1 positivity was also common in those with intermediate or poor risk disease in Checkmate-214 (16).

Neutrophil lymphocyte ratio (NLR) is a measure of inflammation secondary to tumour growth and is the ratio of absolute neutrophil count to absolute lymphocyte count, which has been postulated as a potential biomarker that predicts treatment response to immune checkpoint inhibitor (41, 42). NLR may also be a negative prognostic factor as high NLR is associated with higher risk of death and treatment failure (41, 42). There are small studies to suggest that reduction of NLR pre-treatment and after treatment is associated with improved outcomes in those treated with immune checkpoint inhibitor in advanced RCC (41, 42). However, the value of NLR as a biomarker requires further investigation.

PBRM1 is a possible biomarker, which is a gene that plays a role in remodelling of chromatin (43, 44). PBRM1 mutations occur less frequently in tumours in RCC with high levels of tumour-infiltrating lymphocytes (TILs), which is associated with greater response to nivolumab in an analysis of Checkmate-025 (45). Further, T-cell immunoglobulin-3 (TIM-3) may be found expressed on TILs and contributes to suppression of T-cell mediated immune responses against tumour proliferation hence reduced response to immune checkpoint inhibitors (45, 46). Further investigation into the role of PBRM1 and TIM-3 in predicting treatment response is required. There are currently multiple early phase trials investigating the role of anti-TIM-3 agents in combination with immune checkpoint inhibitors in various cancers including RCC [NCT02817633, NCT03708328].

### Immune Checkpoint Inhibitor Treatment of Metastatic nccRCC

nccRCC is a diverse group of RCCs with various histological subtypes that are vastly different but treated as one group due to rarity of the individual subtypes (2). The most common subtypes of nccRCC include papillary (5 to 10%), chromophobe (5%) and unclassified (< 5%), rarer subtypes including renal medullary, MiT family translocation and SDH-deficient nccRCC all constitute less than 1% of nccRCC (2). There is a lack of evidence to guide treatment of metastatic nccRCC as most clinical trials only included patients with ccRCC. Retrospective studies investigating outcomes in nccRCCs tend to be dominated by patients with papillary and chromophobe subtypes and few with collecting duct, medullary and translocation-associated nccRCC are included as these subtypes are even rarer (2, 46, 47). Overall, nccRCCs demonstrate less response to targeted therapy with anti-VEGF and mTOR inhibitors and has poorer prognosis in survival outcomes when compared to ccRCCs (46, 47). The most recent EAU and NCCN guidelines recommend enrolment of patients with nccRCC onto clinical trials if possible. Targeted therapy such as anti-VEGF TKIs are recommended as first-line treatment of papillary, chromophobe, translocation and unclassified nccRCC whereas platinum-based chemotherapy is recommended for treatment of medullary and collecting duct RCC (29, 30).

nccRCC often have positive PD-L1 expression, which is associated with more advanced disease and poorer prognosis, similar to in ccRCC (48, 49). There is some evidence demonstrating response of nccRCC to immune checkpoint inhibitors but data is limited and mostly retrospective in nature (49, 50). The Keynote-427 phase II trial recruited patients with nccRCC into cohort B of the study, of which 71.5%, 12.7% and 15.8% had papillary, chromophobe and unclassified subtypes respectively (51). This trial demonstrated activity of pembrolizumab in nccRCC with an objective response rate of 24.8% and 81.5% of responders had a durable response of greater than 6 months. Those with papillary and unclassified histology had the highest response rates compared to chromophobe histology (51). It is hypothesised that chromophobe nccRCC are less immunogenic with less immune cell infiltration and therefore have a lower response rate to immune checkpoint inhibitor treatment in comparison to other nccRCC subtypes (52). Checkmate-920 is the first prospective phase III trial to demonstrate the efficacy of nivolumab and ipilimumab in first-line treatment of advanced nccRCC. This trial recruited patients with various nccRCC histological subtypes, including papillary (34.6%), chromophobe (13.5%), unclassified (42.3%) and rarer subtypes including collecting duct (3.8%), medullary (1.9%) and translocation (3.8%) (53). Preliminary results showed an overall objective response rate of 19.6%, progression-free survival of 3.7 months and overall survival of 21.2 months. There were no new safety signals identified with regard to immune-related toxicities when compared to the ccRCC group. SUNIFORECAST is a phase II trial that investigates the efficacy of ipilimumab and nivolumab compared with sunitinb in the first-line treatment of nccRCC and is currently recruiting [NCT03075423]. Data from further prospective studies are required to directly compare the clinical benefit of immune checkpoint inhibitor in the treatment of ccRCC and various subtypes of nccRCC.

## Conclusion

The treatment landscape of metastatic RCC has evolved in the last decade with the rise of immune checkpoint inhibitors in addition to the development of novel TKIs. This has resulted in the improvement of prognosis and survival for patients with metastatic RCC. However, given the rising incidence of metastatic RCC and the lack of evidence to guide treatment of nccRCC, there is a strong need for ongoing research in identification of new biomarkers and development of novel targeted agents to overcome persistent challenges posed by tumour resistance and to guide treatment decisions.

## Author Contributions

IT contributed to the writing and completion of this manuscript. AS contributed to the conception of the research topic and the supervision of this project. All authors contributed to the article and approved the submitted version.

## Conflict of Interest

The authors declare that the research was conducted in the absence of any commercial or financial relationships that could be construed as a potential conflict of interest.

## Publisher’s Note

All claims expressed in this article are solely those of the authors and do not necessarily represent those of their affiliated organizations, or those of the publisher, the editors and the reviewers. Any product that may be evaluated in this article, or claim that may be made by its manufacturer, is not guaranteed or endorsed by the publisher.

## References

[1] Bray F Ferlay J Soerjomataram I Siegel RL Torre LA Jemal A. Global Cancer Statistics 2018: GLOBOCAN Estimates of Incidence and Mortality Worldwide for 36 Cancers in 185 Countries. CA Cancer J Clin (2018) 68(6):394–424. 10.3322/caac.21493 30207593

[2] Padala SA Barsouk A Thandra KC Saginala K Mohammed A Vakiti A. Epidemiology of Renal Cell Carcinoma. World J Oncol; (2020) 11(3):79–87. 10.14740/wjon1279 32494314 PMC7239575

[3] Ljungberg B Campbell S Choi HY Jacqmin D Lee JE Weikert S. The Epidemiology of Renal Cell Carcinoma. Eur Urol (2011) 60(4):615–21. 10.1016/j.eururo.2011.06.049 21741761

[4] Hsieh JJ Purdue MP Signoretti S Swanton C Albiges L Schmidinger M. Renal Cell Carcinoma. Nat Rev Dis Primers (2017) 3:17009. 10.1038/nrdp.2017.9 28276433 PMC5936048

[5] Heng DY Xie W Regan MM Warren M Golshayan AR Sahi C. Prognostic Factors for Overall Survival in Patients With Metastatic Renal Cell Carcinoma Treated With Vascular Endothelial Growth Factor-Targeted Agents: Results From a Large, Multicenter Study. J Clin Oncol (2009) 27(34):5794–9. 10.1200/JCO.2008.21.4809 19826129

[6] Massari F Di Nunno V Guida A Costa Silva CA Derosa L Mollica V. Addition of Primary Metastatic Site on Bone, Brain and Liver to IMDC Criteria in Patients With Metastatic Renal Cell Carcinoma: A Validation Study. Clin Genitourin Cancer (2021) 19(1):32–40. 10.1016/j.clgc.2020.06.003 32694008

[7] Motzer RJ Hutson TE Tomczak P Michaelson MR Bukowski RM Rixe O. Sunitinib *Versus* Interferon Alfa in Metastatic Renal-Cell Carcinoma. N Engl J Med (2007) 356(2):115–24. 10.1056/NEJMoa065044 17215529

[8] Srinivasan R Ricketts CJ Sourbier C Linehan WM. New Strategies in Renal Cell Carcinoma: Targeting the Genetic and Metabolic Basis of Disease. Clin Cancer Res (2015) 21(1):10–7. 10.1168/1078-0432.CCR-13-2993 PMC4795462 25564569

[9] Vachani P George S. VEGF Inhibitors in Renal Cell Carcinoma. Clin Adv Hematol Oncol (2016) 14(12):1016–28. 28212363

[10] Burris HA. Overcoming Acquired Resistance to Anticancer Therapy: Focus on PI3K/AKT/mTOR Pathway. Cancer Chemother Pharmacol (2013) 71(4):829–42. 10.1007/200280-012-2043-3 23377372

[11] Michael A Pandha HS. Renal-Cell Carcinoma: Tumour Markers, T-Cell Epitopes, and Potential for New Therapies. Lancet Oncol (2003) 4(4):215–23. 10.1016/s1470-2045(03)01044-1 12681265

[12] Griffths RW Elkord E Gilham DE Ramani V Clarke N Stern P. Frequency of Regulatory T Cells in Renal Cell Carcinoma Patients and Investigation of Correlation With Survival. Cancer Immunol Immunother (2007) 56(11):1743–53. 10.1007/200262-007-0318-z PMC11030591 17487490

[13] Thompson RH Gillett MD Cheville JC Lohse CM Dong H Webster W. Costimulatory Molecule B7-H1 in Primary and Metastatic Clear Cell Renal Cell Carcinoma. Cancer (2005) 104(10):2084–91. 10.1002/cncr.21470 16208700

[14] Parry RV Chemnitz JM Frauwirth KA Lanfranco AR Braunstein I Kobayashi I. CTLA-4 and PD-1 Receptors Inhibit T-Cell Activation by Distinct Mechanisms. Mol Cell Biol (2005) 25(21):9543–53. 10.1128/MCB.25.21.9543-9553.2005 PMC1265804 16227604

[15] Motzer RJ Tannir NM McDermott DF Frontera OA Melichar B Choueiri T. Nivolumab Plus Ipilimumab *Versus* Sunitinib in Advanced Renal-Cell Carcinoma. N Engl J Med (2018) 378(14):1277–90. 10.1056/NEJMoa1712126 PMC5972549 29562145

[16] Albiges L Tannir NM Burotto M McDermott D Plimack ER Barthelemy P. Nivoumab Plus Ipilimumab *Versus* Sunitinib in Advanced Renal-Cell Carcinoma: Extended 4-Year Follow-Up of the Phase III CheckMate 214 Trial. ESMO Open (2020) 5(6):e001079. 10.1136/esmoopen-2020-001079 33246931 PMC7703447

[17] Motzer RJ Penkov K Haanen J Rini B Albiges L Campbell M. Avelumab Plus Axitinib *Versus* Sunitinib for Advanced Renal-Cell Carcinoma. N Engl J Med (2019) 380(12):1103–15. 10.1056/NEJMoa1816047 PMC6716603 30779531

[18] Choueiri TK Motzer RJ Rini BI Haanen J Campbell MT Venugopal B. Updated Efficacy Results From the JAVELIN Renal 101 Trial: First-Line Avelumab Plus Axitinib *Versus* Sunitinib in Patients With Advanced Renal Cell Carcinoma. Ann Oncol (2020) 31(8):1030–9. 10.1016/j.annonc.2020.04.010 PMC8436592 32339648

[19] Rini BI Plimack ER Stus V Gafanov R Hawkins R Nosov D. Pembrolizumab Plus Axitinib *Versus* Sunitinib for Advanced Renal-Cell Carcinoma. N Engl J Med (2019) 380(12):1116–27. 10.1056/NEJMoa1816714 30779529

[20] Powles T Pilmack ER Soulieres D Waddell T Stus V Gafanov R. Pembrolizumab Plus Axitinib *Versus* Sunitinib Monotherapy as First-Line Treatment of Advanced Renal Cell Carcinoma (KEYNOTE-426): Extended Follow-Up From a Randomised, Open-Label, Phase 3 Trial. Lancet Oncol (2020) 21(12):1563–73. 10.1016/S1470-2045(20)30436-8 33284113

[21] Rini BI Powles T Atkins MB Escudier B McDermott DF Suarez C. Atezolizumab Plus Bevacizumab *Versus* Sunitinib in Patients With Previously Untreated Metastatic Renal Cell Carcinoma (IMmotion151): A Multicentre, Open-Label, Phase 3, Randomised Controlled Trial. Lancet (2019) 393(10189):2404–15. 10.1016/S0140-6736(19)30723-8 31079938

[22] Choueiri TK Powles T Burotto M Escudier B Bourlon MT Zurawski B. Nivolumab plus Cabozantinib versus Sunitinib for Advanced Renal-Cell Carcinoma. N Eng Med (2021) 384:829–41. 10.1056/NEJMoa2026982 PMC8436591 33657295

[23] Motzer R Alekseev B Rha SY Porta C Eto M Powles T. Lenvatinib Plus Pembrolizumab or Everolimus for Advanced Renal Cell Carcinoma. N Engl J Med (2021) 384(14):1289–300. 10.1056/NEJMoa2035716 33616314

[24] Motzer RJ Rini BI McDermott DF Redman BG Kuzel TM Harrison M. Nivolumab for Metastatic Renal Cell Carcinoma: Results of a Randomized Phase II Trial. J Clin Oncol (2015) 33(13):1430–7. 10.1200/JCO.2014.59.0703 PMC4806782 25452452

[25] Motzer RJ Escudier B McDermott DF George S Hammers HJ Srinivas S. Nivolumab *Versus* Everolimus in Advanced Renal-Cell Carcinoma. N Engl J Med (2015) 373(19):1803–13. 10.1056/NEJMoa1510665 PMC5719487 26406148

[26] McDermott DF Lee J Bjarnason GA Larkin J Gafanov RA Kochenderfer M. Open-Label, Single-Arm Phase II Study of Pembrolizumab Monotherapy as First-Line Therapy in Patients With Advanced Clear Cell Renal Cell Carcinoma. J Clin Oncol (2021) 39(9):1020–8. 10.1200/JCO20.02363 PMC8078336 33529051

[27] Roland CL Lynn KD Toombs JE Dineen SP Udugamassoriya DG Brekken R. Cytokine Levels Correlate With Immune Cell Infiltration After Anti-VEGF Therapy in Preclinical Mouse Models of Breast Cancer. PloS One (2009) 4(11):37669. 10.1371/journal.pone.0007669 PMC2766251 19888452

[28] Pilmack ER Powles T Bedke J Pouliot F Stus V Waddell T. Outcomes for Patients in the Pembrolizumab+Axitinib Arm With Advanced Renal Cell Carcinoma (RCC) Who Completed Two Years of Treatment in the Phase III KEYNOTE-426 Study. United States: ASCO GU (2021). Available at: https://meetinglibrary.asco.org/record/195210/abstract.

[29] European Association of Urology (EAU) EAU Guidelines on Renal Cell Carcinoma. (2021) (Accessed April 27 2021).

[30] National Comprehensive Cancer Network (NCCN). NCCN Guidelines Version 4.2021 Kidney Cancer (2021). Available at: https://www.nccn.org/professionals/physician_gls/pdf/kidney.pdf (Accessed 27 April 2021).

[31] Braun DA Bakouny Z Hirsch L Flippot R Van Allen EM Wu C. Beyond Conventional Immune-Checkpoint Inhibition – Novel Immunotherapies for Renal Cell Carcinoma. Nat Rev Clin Oncol (2021) 18(4):199–214. 10.1038/s41571-020-00455-z 33437048 PMC8317018

[32] Motzer R Eisen T Hutson T Szczylik C Krygowski M Strahs A. Overall Survival Results From a Phase III Study of Tivozanib Hydrochloride *Versus* Sorafenib in Patients With Renal Cell Carcinoma. J Clin Oncol (2013) 31(6):350–0. 10.1200/jco.2013.31.6_suppl.350

[33] Rini B Pal S Escudier B Atkins MB Hutson T Porta C. Tivozanib *Versus* Sorafenib in Patients With Advanced Renal Cell Xarcinoma (TIVO-3): A Phase 3, Multicentre, Randomised, Controlled, Open-Label Study. Lancet Oncol (2020) 21(1):95–104. 10.1016/S1470-2045(19)30735-1 31810797

[34] Albiges L Barthelemy P Gross-Goupil M Negrier S Needle M Escudier B. TiNivo: Safety and Efficacy of Tivozanib-Nivolumab Combination Therapy in Patients With Metastatic Renal Cell Carcinoma. Ann Oncol (2021) 32(1):97–102. 10.1016/j.annonc.2020.09.021 33010459

[35] Aveo Oncology. FOTIVDA (Tivozanib) in Combination With OPDIVO (Nivolumab) in Pivotal Phase 3 TiNivo-2 Trial in IO Relapsed Renal Cell Carcinoma (2021). Available at: http://investor.aveooncology.com/news-releases/news-release-details/aveo-oncology-announces-collaboration-bristol-myers-squibb (Accessed 22 July 2021).

[36] Choueiri TK Bauer TM Papadopoulos KP Plimack ER Merchan JR Merchan J. Inhibition of Hypoxia-Inducible Factor-2α in Renal Cell Carcinoma With Belzutifan: A Phase 1 Trial and Biomarker Analysis. Nat Med (2021) 27(5):802–5. 10.1038/s41591-021-01324-7 PMC9128828 33888901

[37] Munn DH Mellor AL. Indoloeamine 2,3-Dioxygenase and Tumour-Induced Tolerance. J Clin Invest (2007) 117(5):1147–54. 10.1172/JCI31178 PMC1857253 17476344

[38] Mitchell T Hamid O Smith DC Bauer TM Wasser JS Olszanski A. Epacadostat Plus Pembrolizumab in Patients With Advanced Solid Tumours: Phase I Results From a Multicentre, Open-Label Phase I/II Trial (ECHO-202/KEYNOTE-037). J Clin Oncol (2018) 36(32):3223–30. 10.1200/JCO.2018.78.9602 PMC6225502 30265610

[39] Long GV Dummer R Hamid O Gajewski T Caglevic C Dalle S. Epacadostat Plus Pembrolizumab *Versus* Placebo Plus Pembrolizumab in Patients in Unresectable or Metastatic Melanoma (ECHO-301/ KEYNOTE-252): A Phase 3, Randomised, Double-Blind Study. Lancet Oncol (2019) 20(8):1083–97. 10.1016/S1470-2045(19)30274-8 31221619

[40] Iacovelli R Nole F Verri E Renne G Paglino C Santoni M. Prognostic Role of PD-L1 Expression in Renal Cell Carcinoma. A Systematic Review and Meta-Analysis. Target Oncol (2016) 11(2):143–8. 10.1007/s11523-015-0392-7 26429561

[41] Basu A Phone A Bice T Sweeney P Acharya L Suri Y. Change in Neutrophil to Lymphocyte Ratio (NLR) as a Predictor of Treatment Failure in Renal Cell Carcinoma Patients: Analysis of the IROC (Investigating RCC Outcomes) Cohort. J Clin Oncol (2021) 39(6):344–4. 10.1200/JCO.2021.39.6_suppl.344

[42] Lalani A Xie W Martini D Steinharter J Norton C. Change in Neutrophil-To-Lymphocyte Ratio (NLR) in Response to Immune Checkpoint Blockade for Metastatic Renal Cell Carcinoma. J Immunother Cancer (2018) 6(1):5. 10.1186/s40425-018-0315-0 29353553 PMC5776777

[43] Miao D Margolis CA Gao W Voss MH Li W Martini D. Genomic Correlates of Response to Immune Checkpoint Therapies in Clear Cell Renal Cell Carcinoma. Science (2018) 359(6377):801–6. 10.1126/science.aan5951 PMC6035749 29301960

[44] Bruan D Hou Y Bakouny Z Ficial M Miriam Sant’Angelo Forman J. Interplay of Somatic Alterations and Immune Infiltration Modulates Response to PD-1 Blockade in Advanced Clear Cell Renal Cell Carcinoma. Nat Med (2020) 26(6):909–18. 10.1037/s41591-020-0839 PMC7499153 32472114

[45] Ficial M Jegede O Sant’Angelo M Moreno S Braun D Wind-Rotolo M. Evaluation of Predictive Biomarkers for Nivolumab in Patients With Metastatic Clear Cell Renal Cell Carcinoma (mccRCC) From the CheckMate-025 Trial. J Clin Oncol (2020) 38(15):5023. 10.1200/JCO.2020.38.15_suppl.5023

[46] Sakuishi K Ngiow SF Sullivan JM Teng MW Kuchroo VK. TIM3+FOXP3+ Regulatory T Cells Are Tissue-Specific Promoters of T-Cell Dysfunction in Cancer. Oncoimmunology (2013) 2(4):e23849. 10.4161/onci.23849 23734331 PMC3654601

[47] Fernandez-Pello S Hofmann F Tahbaz R Marconi L Lam T Albiges L. A Systematic Review and Meta-Analysis Comparing the Effectiveness and Adverse Effects of Different Systemic Treatments for Non-Clear Cell Renal Cell Carcinoma. Eur Urol (2017) 71(3):426–36. 10.1016/j.eururo.2016.11.020 27939075

[48] Kroeger N Xie W Lee J Bjarnason G Knox JJ Mackenzie M. Metastatic non-Clear Cell Renal Cell Carcinoma Treated With Targeted Therapy Agents: Characterisation of Survival Outcome and Application of the International mRCC Database Consortium Criteria. Cancer (2013) 119(6):2999–3006. 10.1002/cncr.28151 23696129 PMC3934562

[49] Mckay RR Bosse D Xie W Wankowicz SA Flaifel A Brandao R. The Clinical Activity of PD-1/PD-L1 Inhibitors in Metastatic Non-Clear Cell Renal Cell Carcinoma. Cancer Immunol Res (2018) 6(7):758–65. 10.1158/2326-6066.CIR-17-0475 PMC6712567 29748390

[50] Choueiri TK Fay AP Gray KP Ho TH Albiges L Bellmunt J. PD-L1 Expression in Nonclear-Cell Renal Cell Carcinoma. Ann Oncol (2014) 25(11):2178–84. 10.1093/annonc/mdu445 PMC4288138 25193987

[51] McDermott D Lee J Ziobro M Suarez C Langiewicz P Matveev V. Open-Label, Single-Arm, Phase II Study of Pembrolizumab Monotherapy as First-Line Therapy in Patients With Advanced Non-Clear Cell Renal Cell Carcinoma. J Clin Oncol (2021) 39(9):1029–39. 10.1200/JCO.20.02365 PMC8078262 33529058

[52] Zoumpourlis P Genovese G Tannir NM Msaouel P. Systemic Therapies for the Manaegment of Non-Clear Cell Renal Cell Carcinoma: What Works, What Doesn’t, and What the Future Holds. Clin Genitourin Cancer (2021) 19(2):103–16. 10.1016/j.clgc.2020.11.005 PMC8169717 33358151

[53] Tykodi S Gordan L Alter R Arrowsmith E Harrison MR Percent I. Nivolumab Plus Ipilimumab in Patients With Advanced non-Clear Cell Renal Cell Carcinoma (nccRCC): Safety and Efficacy From CheckMate 920. J Clin Oncol (2021) 39(6):309–9. 10.1200/JCO.2021.39.6_suppl.309

